# Incidence and Course of Joint Inflammation Associated with Inflammatory Bowel Disease in Patients Undergoing Treatment with Vedolizumab/Ustekinumab: The VEDUSTAR Study

**DOI:** 10.3390/jcm13041076

**Published:** 2024-02-14

**Authors:** Irene Gonzalez Diaz, Mariana Gutierrez Riart, Maria Dolores Martin-Arranz, Chamaida Plasencia Rodriguez, Cristina Suarez Ferrer, on behalf of VEDUSTAR Research Team

**Affiliations:** 1 Gastroenterology Department, La Paz University Hospital, 28046 Madrid, Spain; cristinajulia.suarez@salud.madrid.org; 2 Rheumatology Department, La Paz University Hospital, 28046 Madrid, Spain; mariana.gutierrez@salud.madrid.org (M.G.R.); chamaida.plasencia@salud.madrid.org (C.P.R.); 3 Hospital La Paz Institute for Health Research, La Paz University Hospital, 28046 Madrid, Spain; 4 Faculty of Medicine, Universidad Autónoma, 28046 Madrid, Spain

**Keywords:** spondyloarthritis, joint symptoms, VDZ, UST, intestinal activity

## Abstract

Background: The role of ustekinumab (UST) and vedolizumab (VDZ) in the extraintestinal joint manifestations of inflammatory bowel disease (IBD) remain unclear, and most existing studies are retrospective. The aim of this prospective study was to analyze the incidence of new-onset joint disease or the worsening of pre-existing IBD-associated joint disease in patients treated with UST and VDZ. Methods: The study population comprised IBD patients with previous spondyloarthritis (SpA) or new-onset arthropathy undergoing treatment with VDZ or UST. Results: Eighty patients were referred to rheumatology because of previous SpA or onset of symptoms. Most patients (90%) were anti-TNF experienced. Two patients with previous SpA (2/22; 9%) experienced a flare-up (one with UST and one with VDZ), and two patients with VDZ developed SpA during follow-up (2/58; 3%). Only one of these four patients did not have gastrointestinal symptoms, and VDZ was discontinued because of joint symptoms. The other three patients had concomitant intestinal activity, and treatment was not discontinued. Conclusion: Our experience shows that treatment with UST and VDZ did not worsen joint disease in patients with SpA. Most remained stable or improved. In addition, poor control of IBD in patients with joint flare-ups could be the main cause of worsening SpA.

## 1. Introduction

Joint involvement is a common extraintestinal manifestation (EIM) of inflammatory bowel disease (IBD). It can be axial and/or peripheral and can occur parallel to or independently of luminal activity [1]. Its prevalence varies according to the literature. In our setting, it affects approximately one-third of patients [2,3,4]. The prevalence is more frequent in patients with Crohn’s disease (CD), 20–40%, and somewhat lower in patients with ulcerative colitis (UC), 5–10% [5]. Regarding the type of arthritis, the peripheral form affects 2.8–31% of patients and the axial form 2–45.5% [6].

The demonstrated efficacy of tumor necrosis factor-alpha antagonists (anti-TNF) in various randomized clinical trials makes them the treatment of choice for affected patients [7,8,9,10]. Biological treatments with new therapeutic targets other than anti-TNFs, such as ustekinumab (UST) and vedolizumab (VDZ), have been available for some years and have revolutionized patient management in terms of control of intestinal disease. However, the efficacy of these drugs against EIMs remains controversial.

VDZ is a monoclonal anti-integrin alpha4-beta7 antibody that has no systemic effect, but rather prevents leukocyte migration at the intestinal level. It is indicated for both CD and UC [11,12]. Its efficacy in EIM is controversial because of its selective effect in the intestine. To date, no clinical trials have been conducted to assess the efficacy of VDZ in joint involvement associated with IBD, and available evidence is conflicting. Some studies report an improvement in joint symptoms [13,14], whereas in others, a worsening of joint symptoms led to discontinuation of treatment [15].

UST is a human monoclonal antibody that blocks the p40 subunit of interleukin-12 (IL-12) and interleukin-23 (IL-23). It has been approved for CD and, more recently, for UC [16,17]. IL-23 has been involved in the pathogenesis of joint pathologies, such as ankylosing spondylitis (AS), spondyloarthritis (SpA), and psoriasis. For this reason, UST could be effective in the management of these diseases. Overexpression of IL-23 in the subchondral bone marrow and fibrous tissue has been shown to replace bone marrow in the facet joints of patients with AS [18], and a polymorphism within the IL-23 receptor could affect sensitivity to developing AS [19]. Elsewhere, IL-23 blockade has proven effective in CD and psoriasis [20,21,22,23], with clinical and radiographic remission reported in randomized trials [24,25,26]. Improvements were observed in various parameters, namely clinical findings, biochemistry values (C-reactive protein [CRP]), and radiological data (magnetic resonance imaging [MRI]) [27]. For SpA, elevated levels of IL-23 have also been observed in the synovial tissue and peripheral blood of these patients [28]. UST has demonstrated efficacy in patients with peripheral SpA and in patients with an inadequate response to anti-TNF agents in a phase II clinical trial [29]. However, phase III clinical trials have revealed no significant differences in the clinical and biochemical response compared with placebo [30].

Robust evidence from real-world clinical practice is lacking for both VDZ and UST. 

We performed a prospective study to analyze the incidence of previous SpA flare-ups and the onset of undiagnosed SpA in IBD patients undergoing treatment with UST and VDZ.

## 2. Materials and Methods

### 2.1. Study Design

We performed a prospective study at a single tertiary center from February to November 2022. This study was carried out in accordance with good clinical practice guidelines as outlined in the declaration of Helsinki and approved by the ethics committee of La Paz University Hospital (PI-4965/2021). Informed consent was obtained from all subjects involved in the study. The study population comprised patients with IBD who were undergoing treatment with UST or VDZ. The inclusion criteria were age ≥18 years and a diagnosis of CD or UC actively treated with UST or VDZ according to the standard protocol as set out in the summary of product characteristics. The exclusion criteria were absence of clinical data at the baseline visit for the UST or VDZ study and a confirmed diagnosis or suspicion of psoriatic arthritis.

**Figure 1:**
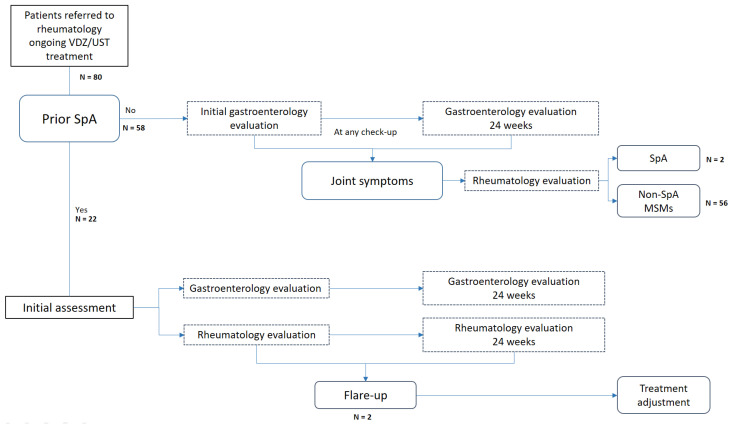
Flowchart of patient referral to the rheumatology department according to the study design. VDZ: vedolizumab; UST: ustekinumab; SpA: spondyloarthritis; MSMs: musculoskeletal manifestations.

All patients with a prior diagnosis of SpA who were actively undergoing treatment with UST or VDZ were referred to rheumatology, as were those who presented joint symptoms (arthralgia, arthritis, low back pain, or enthesitis pain) without a previous diagnosis during the study period (see Figure 1). Patients were assessed in the gastroenterology and rheumatology departments within <72 h. The rheumatology department conducted the initial evaluation and scheduled subsequent visits according to the specialist’s clinical suspicion to confirm/exclude a diagnosis of SpA (onset or flare-up) associated with IBD.

The frequency of and reasons for drug discontinuation during follow-up were also documented.

### 2.2. Patients and Variables

Sociodemographic data and treatments are detailed in Table 1.

In the gastroenterology assessment, clinical activity was assessed according to validated indices (Harvey–Bradshaw index [HBI] for CD and partial Mayo score for UC) at baseline and at 6 months, with patients classified as being in clinical remission or having experienced a mild, moderate, or severe flare. Biochemical response markers (CRP and fecal calprotectin [FC]), were recorded, as were imaging results (endoscopy, ultrasound) if available.

During the rheumatology assessment, the presence of prior SpA or other joint involvement at the start of treatment with VDZ or UST was recorded. This included the type (AS, axial non-radiographic SpA, peripheral SpA), the main symptomatic component (axial, peripheral, or enthesitis), clinical activity (low activity or remission defined as ASDAS > 2.1 or BASDAI < 4), biochemistry values (CRP and erythrocyte sedimentation rate), and radiology findings (sacroiliac joint MRI or ultrasound) if available.

A flare-up of SpA was defined as a clinically relevant worsening at the joint level according to the rheumatologist’s criteria requiring a specific therapeutic approach. Similarly, the onset of SpA was defined as the first manifestation of joint symptoms in patients diagnosed with SpA according to the treating rheumatologist, irrespective of whether they met the classification criteria for this disease while they were being treated with VDZ or UST.

### 2.3. Statistical Analysis

We performed a descriptive analysis of the sample and calculated the mean (standard deviation) and median (interquartile range) for demographic, analytical, and quantitative clinical variables.

Means were compared using the *t*-test when the variables followed a normal distribution (verified using the Shapiro–Wilk test).

## 3. Results

### 3.1. Demographic Characteristics

**Figure 2:**
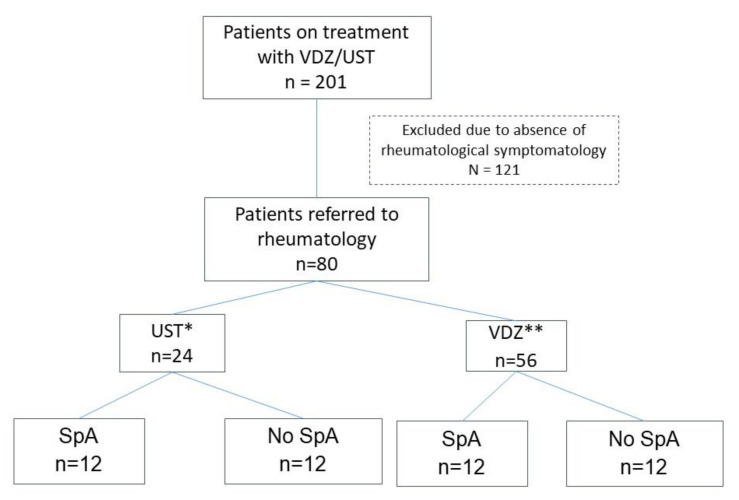
Patients referred to rheumatology ** VDZ: vedolizumab; * UST: ustekinumab; SpA: spondyloarthritis.

At the time of the study, 201 patients were receiving treatment with UST (112) and VDZ (89). Eighty were referred to the rheumatology department with a history of SpA or because they had developed joint symptoms. Of these, 56 (70%) were taking VDZ, and 24 (30%) were taking UST (see Figure 2). Only one patient had recently initiated therapy with VDZ at the beginning of the study. The remaining patients had been receiving treatment with VDZ/UST for more than 6 months.

The sociodemographic characteristics of these patients are summarized in Table 1.

Of the 80 patients referred to the rheumatology department, 24 (30%) were classified as having SpA; of these, 22 (92%) had previously been diagnosed, and in 2 (8%), the first manifestations were during treatment with VDZ. The remaining 56 (70%) patients were diagnosed with other rheumatological conditions (see Table 2).

Of the total cohort evaluated, only seven patients (8%) had not been previously treated with biologics. Most had previously received biologics (58 [73%]), mainly anti-TNF agents (73%). On the other hand, almost half of the patients received UST or VDZ as monotherapy (38 [48%]). Of the remaining 42 patients, 52% received concomitant disease-modifying antirheumatic drugs (DMARDs), with sulfasalazine being the most frequent in patients with SpA (4% vs. 0%). Combinations of biologic agents (VDZ or UST + anti-TNF) were only used in two patients within the SpA group.

Of all the patients with SpA, 11 (46.4%) had only peripheral involvement, 7 (29%) had only axial involvement, and 6 (25%) had mixed involvement (axial and peripheral).

### 3.2. Gastrointestinal Activity of Referred Patients

At the baseline visit, most patients (62.5%) were in clinical remission, six (25%) had mild activity, one (4%) had moderate activity, and two (8%) had severe activity according to clinical activity indices. Regarding biochemical response markers, baseline CRP was 6.06 ± 10.81 mg/L, and FC was 477 ± 701.98 μg/g. Additionally, seven patients (8.8%) underwent intestinal ultrasound at the baseline visit; this revealed mild activity in two (6.8%), moderate activity in one (3.4%), and severe activity in four (13.8%).

A slight exacerbation of gastrointestinal disease was observed at 6 months (see Figure 1); 13 patients (54%) were in clinical remission, 5 (21%) experienced a moderate flare, and 5 (21%) experienced a severe flare. CRP at 6 months was 8.90 ± 11.9 mg/L, and FC was 624.51 ± 1125.23 µg/g (see Figure 2). Six patients (7.5%) underwent intestinal ultrasound, which revealed no activity in one (3.4%), mild activity in two (6.8%), and severe activity in three (10.3%).

### 3.3. Rheumatologic Activity of Referred Patients

The most frequent rheumatologic manifestation in patients with prior SpA was peripheral involvement. This was peripheral in only 11 (50%) and mixed axial and peripheral in 4 (18%). However, in patients whose SpA first manifested during treatment with UST or VDZ, mixed involvement predominated.

Joint activity remained stable or even improved in most of the patients with prior SpA (see Table 3). Axial involvement was observed in only two of these patients (9%); one was being treated with UST and the other with VDZ. Both had experienced moderate flare-ups of ileocolitis (HBI, 8 and 9 points; respectively) with CRP > 2.5 mg/L and FC > 600 µg/g. In the patient taking VDZ, the flare-up was controlled by adding an anti-TNF agent, and in the patient with UST, symptomatic treatment was adjusted (selective cyclooxygenase-2 inhibitors [COX2]).

SpA first manifested with mixed involvement (axial and peripheral) in two patients (3.6%) taking VDZ. Both had ileocolitis. One patient experienced a moderate flare (HBI, 13 points; CRP, 16 mg/L; and FC, 97 µg/g). Biological treatment was switched to an anti-interleukin 23 agent (anti–IL-23) to control gastrointestinal and rheumatologic symptoms. The other patient had no gastrointestinal symptoms (HBI, 3 points; CRP, 0.5 mg/L; and FC, 55 µg/g). An anti-TNF agent was started, owing to the predominance of joint symptoms, and treatment with concomitant DMARDs was adjusted.

A multivariate analysis of SpA flare-ups (see Table 4) with *p* < 0.20 as significant revealed the variables associated with an increased risk of joint worsening to be smoking (OR 1.9 [0.87–4.13]; *p* = 0.10) and the number of previous biologics (OR 2.32 [0.8–6.73]; *p* = 0.12). 

### 3.4. Discontinuation of UST or VDZ

VDZ or UST was discontinued during follow-up in 14/80 (17.5%) patients. This was because of poor control of gastrointestinal symptoms in 11/14 (78.6%), tumor in 2/14 (14.2%), and poor control of joint symptoms in 1/14 (7%). Of the total cohort, 1.3% discontinued treatment.

## 4. Discussion

The role of UST and VDZ in the control of SpA is uncertain, with evidence mainly from retrospective studies. Our prospective analysis did not reveal more frequent worsening of joint involvement in patients treated with UST or VDZ. In addition, gastrointestinal activity was poorly controlled in patients with joint symptoms.

Within our cohort, a higher proportion of patients receiving VDZ were referred to rheumatology for joint involvement than patients receiving UST (56 [70%] vs. 24 [30%]). Patients were classified as SpA and non-SpA, with no significant differences in sociodemographic variables. It is important to highlight that the criteria for referral to rheumatology included not only arthritis but also the presence of arthralgia, low back pain, or enthesitis pain in patients with IBD, regardless of whether or not the pain was associated with inflammation. Consequently, patients referred to rheumatology may have included a high proportion of non-SpA cases (70%).

The most frequent joint involvement was peripheral in SpA patients, with a frequency of 50%, which was similar to that reported in other studies where peripheral involvement predominated over axial involvement [15,31,32]. However, this was not the same in cases of the first manifestation of the disease, where the mixed form (axial and peripheral) predominated.

Evidence suggests that VDZ is associated with a higher incidence of joint symptoms, although most studies are retrospective [33,34,35]. However, other authors do not observe this association and even report an improvement in joint manifestations in the long term or no significant differences in the incidence of arthralgia in patients treated with VDZ [11,13,14,36]. Furthermore, the authors attribute this effect to the intestinal remission observed, since we know that VDZ acts selectively without exerting a systemic effect [14,36,37]. It seems that concurrent gastrointestinal activity is a major risk factor for the worsening of joint symptoms [38,39].

Joint symptoms were well controlled in most patients treated with VDZ in our study. Only one patient with prior SpA (5%) experienced a flare-up and simultaneously had intestinal activity. However, we cannot establish a statistical association between joint and intestinal activity, owing to the sample size.

The efficacy of UST in arthralgia seems superior to that of VDZ. UST has been shown to slow clinical and radiological progression in psoriatic arthritis [4,23,24,40]. This is explained by the overexpression of the Il-12 and IL-23 subunit p40 in plaque psoriasis [41,42]. However, evidence in non-psoriatic joint involvement is more limited. IL-12/Th17 has been implicated in the pathogenesis of SpA in preclinical studies [18,19,20], with an improvement in clinical, biochemical (CRP), and radiological (MRI) parameters [26]. 

In observational studies, remission of joint involvement with UST ranged from 32.4% to 82.2% [43,44]. However, clinical trials have not demonstrated the effectiveness of UST for axial SpA [30]. In our cohort, only one patient with UST (4%) developed a joint flare, while the rest remained stable. Most (70%) were receiving UST in monotherapy without the need for DMARDs.

Concerning the onset of SpA, it is not uncommon to encounter new arthralgia in patients initiating VDZ or UST, as reported in the literature. With VDZ, rates of up to 14%–15% have been reported [14,45]. Arthralgia can manifest as frequent adverse effects [11,46]. In the case of UST, the rates range from 7.3% to 16.7% [47,48,49].

In our study, two patients (3.6%) developed SpA under treatment with VDZ and thiopurine, respectively. Only one patient remained asymptomatic in terms of gastrointestinal activity, requiring a switch to an anti-TNF agent owing to joint symptoms. Treatment for the other patient, who had poorly controlled intestinal symptoms, was switched to an anti–IL-23 agent. This low incidence of onset may also be attributed to the fact that over 50% of VDZ-treated patients received concurrent DMARDs.

Nevertheless, newly occurring arthralgia does not usually lead to treatment discontinuation [50]. Discontinuation rates in studies are generally low, ranging from 0.4% to 9.5% [15,50,51]. In our cohort, treatment was only discontinued owing to poorly controlled joint symptoms in one patient taking VDZ (1.3%). Additionally, 49% of patients who did not fulfill the criteria for SpA experienced nonspecific arthralgia that did not lead to a change in treatment.

A recent multicenter study comparing both drugs (584 patients receiving VDZ and 327 receiving UST) concluded that worsening of arthropathy is similar in both groups (35.9% for VDZ vs. 22.5% for UST, with no significant differences) and remains comparable at 1 year of follow-up. Onset of arthropathy is comparable at baseline (2% vs. 2.4%, *p* = 0.69) and at 6 months between both groups (0.8% in VDZ and 0.4% in UST, *p* = 0.663). The authors found that the risk of developing arthralgia in the first 6 months of treatment was only higher in the VDZ group and that this risk disappeared at 2 years of treatment [47].

Ferretti et al. performed a multicenter retrospective study of 1182 patients, in whom they compared the rate of de novo EIMs and the worsening of pre-existing ones between VDZ and other non-selective biologic therapies, including UST. The authors observed that the rate of de novo EIMs was higher for the VDZ group, with significant differences (6.6% vs. 3.5%, *p* = 0.02), especially in rheumatologic EIMs. They also found more frequent worsening of pre-existing EIMs, albeit without significant differences (15.5% vs. 7.3%, *p* = 0.08). However, with no need to change treatment, the clinical course was similar in both groups. These studies point to a slightly higher risk of developing EIMs with selective biologic therapies, although it is true that most patients had previously been treated with anti-TNF agents, which could have unmasked the EIM [31,32,38].

In any case, the long-term course of joint disease is similar with both UST and VDZ, and there is no need to discontinue treatment [50,51]. In our study, more patients taking VDZ were referred for musculoskeletal joint disease (70%) than those taking UST. However, the SpA rate was low, with no significant differences.

Finally, other factors associated with the risk of de novo joint-related EIMs in these patients [38,47,52] include previous anti-TNF therapy [38], disease duration [53], and intestinal activity [5,13,36]. Our multivariate analysis revealed a higher risk of developing SpA in smokers (OR: 1.9 [0.87–4.13]; *p* = 0.10) and patients who have already taken a large number of biologics (OR: 2.32 [0.8–6.73]; *p* = 0.12).

Our study is subject to limitations, including its small sample size, which likely prevents us from finding significant differences and fully comparing the treatment groups. Follow-up lasted only 6 months, and baseline clinical status did not necessarily reflect the condition at the beginning of treatment with VDZ/UST (this would have been the optimal situation). However, this study’s strengths are its prospective design and the fact that the diagnoses were confirmed or ruled out by rheumatologists, who conducted a comprehensive assessment in conjunction with gastroenterologists.

## 5. Conclusions

In our experience, treatment with UST and VDZ did not lead to worsening of joint disease in patients with SpA. Instead, joint disease remained mostly stable or improved. Patients with joint flares also had poorly controlled intestinal symptoms, suggesting that the worsening of joint symptoms was secondary to gastrointestinal activity. These drugs could be considered in cases where joint-related EIMs occur simultaneously with luminal activity. However, before initiating biological treatment in affected patients, a multidisciplinary evaluation is crucial to choosing the most appropriate agent. 

## Figures and Tables

**Table 1:** Baseline characteristics of patients.

	Total (*n* = 80)	SpA (*n* = 24)	Non-SpA (*n* = 56)	*p*-Value
Sociodemographic Variables				
Mean age (years) *	52.16 ± 14.3	54.9 ± 13.1	36.93 ± 18.76	0.4
Disease duration *	14.28 ± 9.4	13.29 ± 7.35	14 ± 10.18	0.2
Sex				
Male	36 (45%)	10 (42%)	26 (46%)	0.695
Female	44 (55%)	14 (58%)	30 (54%)	
IBD type				
UC	27 (34%)	7 (29%)	20 (36%)	0.278
CD	52 (65%)	16 (67%)	36 (64%)	
Indeterminate colitis	1 (1%)	1 (4%)	0 (0%)	
Smoking status				
Never	38 (47.5%)	10 (42%)	28 (50%)	0.519
Former smoker	30 (37.5%)	11 (46%)	19 (34%)	
Active smoker	12 (15%)	3 (13%)	9 (16%)	
Specific Characteristics of IBD				
Type of CD				
Ileitis	24 (30%)	6 (25%)	18 (32%)	0.823
Ileocolitis	19 (24%)	6 (25%)	13 (23%)	
Colitis	2 (3%)	1 (4%)	1 (2%)	
Jejunoileitis	7 (9%)	2 (8,3%)	5 (9%)	
Type of UC				
Left-sided	9 (11%)	2 (8%)	7 (12.5%)	0.940
Pancolitis	18 (23%)	4 (17%)	14 (25%)	
CD phenotype				
Inflammatory	16 (20%)	5 (33%)	11 (20%)	0.577
Stricturing	17 (21%)	6 (40%)	11 (20%)	
Fistulizing	18 (22.5%)	4 (27%)	14 (25%)	
Immune-mediated inflammatory diseases	19 (24%)	11 (46%)	8 (14%)	0.001
Perianal disease (PD)	15 (19%)	3 (10.3%)	12 (21%)	0.461
VDZ/UST Treatment				
VDZ	56 (70%)	12 (50%)	44 (79%)	0.011
UST	24 (30%)	12 (50%)	12 (21%)	
HLA-B27 known status	48 (60%)			
Positive	5 (6%)	4 (17%)	1 (2%)	0.156
Negative	43 (54%)	20 (83%)	23 (41%)	
HLA-B27 unknown status	32 (40%)	0 (0%)	32 (57%)	
Previous Biological Treatment				
1 biologic	24 (30%)	5 (21%)	19 (34%)	0.086
2 biologics	34 (43%)	8 (33%)	26 (46%)	
≥3 biologics	15 (19%)	9 (38%)	6 (11%)	
Biologic-naïve	7 (8%)	2 (8%)	5 (9%)	
Concomitant DMARDs				
Thiopurines	30 (37%)	5 (21%)	25 (45%)	0.006
Methotrexate	4 (5%)	1 (4%)	3 (5%)	
Sulfasalazine	4 (5%)	4 (17%)	0 (0%)	
Mesalazine	4 (5%)	1 (4%)	3 (5%)	
Monotherapy	38 (48%)	13 (54%)	25 (45%)	
Corticosteroid use	3 (4%)	1 (4%)	2 (4%)	0.441
Combined Biological Therapy				
VDZ + anti-TNF	1 (1%)	1 (4%)	0 (0%)	-
UST + anti-TNF	1 (1%)	1 (4%)	0 (0%)	

UC: ulcerative colitis; CD: Crohn’s disease; VDZ: vedolizumab; UST: ustekinumab; HLA-B27: human leukocyte antigen B27; DMARDs: disease-modifying antirheumatic drugs; anti-TNF: tumor necrosis factor-alpha antagonists. * Data expressed as mean ± standard deviation.

**Table 2:** Musculoskeletal diseases.

Type	Frequency
Spondyloarthritis (SpA) (n = 24)	
Previous SpA (n = 22):	
Axial	7 (32%)
Peripheral	11 (50%)
Mixed: axial and peripheral	4 (18%)
Onset of SpA(n = 2):	
Axial	0 (0%)
Peripheral	0 (0%)
Mixed	2 (100%)
Non-SpA (n = 56)	
Osteoarthritis	8 (14%)
Microcrystalline arthritis	2 (3%)
Connective tissue diseases	3 (6%)
Non-inflammatory low back pain	4 (7%)
Non-inflammatory joint pain	39 (70%)

**Table 3:** Classification of joint activity in patients with prior SpA taking UST or VDZ.

	Total (n = 22)	UST (n = 12)	VDZ (n = 10)
Improvement	5 (23%)	5 (42%)	0 (0%)
Stability	15 (68%)	6 (50%)	9 (90%)
Flare-up	2 (9%)	1 (8%)	1 (10%)

VDZ: vedolizumab; UST: ustekinumab.

**Table 4:** Multivariate regression model assessing risk factors for SpA flare-ups.

	Odds Ratio	Std. Error	*p*-Value	95% Confidence Interval
Active smoker	1.9	0.73	0.10	[0.87–4.13]
HLA-B27	1.9	2.66	0.64	[0.12–29.26]
Number of biologic treatments	2.3	1.26	0.12	[0.80–6.73]
VDZ	1.6	3.35	0.82	[0.03–96.48]
UST	0.6	0.58	0.57	[0.14–2.98]

HLA-B27: human leukocyte antigen B27; VDZ: vedolizumab; UST: ustekinumab; SpA: spondyloarthritis.

## Data Availability

The data presented in this study are available on request from the corresponding author.
